# Docetaxel Skin Exposure and Micronucleation Contributes to Skin Toxicity Caused by CPC634

**DOI:** 10.3390/cancers13153741

**Published:** 2021-07-26

**Authors:** Florence Atrafi, Ruben A. G. van Eerden, Stijn L. W. Koolen, Peter de Bruijn, Cristianne J. F. Rijcken, Rob Hanssen, Ferry A. L. M. Eskens, Martijn P. Lolkema, Esther Oomen-de Hoop, Jeffrey Damman, Ron H. J. Mathijssen

**Affiliations:** 1 Department of Medical Oncology, Erasmus MC Cancer Institute, 3015 GD Rotterdam, The Netherlands; r.vaneerden@erasmusmc.nl (R.A.G.v.E.); s.koolen@erasmusmc.nl (S.L.W.K.); p.debruijn@erasmusmc.nl (P.d.B.); f.eskens@erasmusmc.nl (F.A.L.M.E.); m.lolkema@erasmusmc.nl (M.P.L.); e.oomen-dehoop@erasmusmc.nl (E.O.-d.H.); a.mathijssen@erasmusmc.nl (R.H.J.M.); 2 Department of Hospital Pharmacy, Erasmus MC, 3015 GD Rotterdam, The Netherlands; 3 Cristal Therapeutics, 6229 EV Maastricht, The Netherlands; cristianne.rijcken@cristaltherapeutics.com (C.J.F.R.); rob.hanssen@cristaltherapeutics.com (R.H.); 4 Department of Pathology, Erasmus MC, 3015 GD Rotterdam, The Netherlands; j.damman@erasmusmc.nl

**Keywords:** nanoparticles, toxicity, docetaxel, pharmacokinetics, pharmacodynamics, chemotherapy, skin

## Abstract

**Simple Summary:**

CPC634 is a nanoparticle entrapping docetaxel that is associated with skin toxicity that resembles conventional docetaxel-related skin toxicity. In this randomised cross-over study, the cutaneous pharmacokinetics and pharmacodynamics of docetaxel and CPC634 were compared to unravel the mechanisms behind the cutaneous toxicity. The total docetaxel concentration in the skin was almost four-fold higher after CPC634 administration compared to conventional docetaxel. Both CPC634 and conventional docetaxel administration resulted in anti-mitotic effects in the skin such as micronucleation. Micronucleation can induce an inflammatory reaction, which could lead to skin toxicity.

**Abstract:**

Docetaxel entrapped nanoparticle CPC634 is associated with dose-related skin toxicity that resembles conventional docetaxel (Cd)-related skin toxicity. This study compared the cutaneous pharmacokinetics and pharmacodynamics of docetaxel and CPC634. In this randomised cross-over study, patients with solid tumours received one cycle of CPC634 and Cd (both at 75 mg/m^2^). Skin biopsies were taken at baseline and at day 8 of both cycles. Released and total docetaxel (released docetaxel plus entrapped docetaxel) concentrations and histopathological changes in the skin biopsies were evaluated. Twenty patients underwent paired skin biopsies for pharmacokinetic analysis and 10 patients had biopsies available for histopathological assessment. The total skin docetaxel concentration was 369% (95%CI: 229% to 569%, *p* < 0.001) higher after CPC634 administration compared to Cd while the released docetaxel concentrations were not statistically different (95%CI: −9% to 63%, *p* = 0.169). The CPC634 released docetaxel concentration in the skin was positively correlated with plasma concentrations (Pearson’s correlation 0.48, *p* = 0.03). Histopathological examination revealed increased apoptosis, mitotic cells with nuclear atypia, and micronucleation with an enhanced Ki-67 index for both compounds. In conclusion, both CPC634 and Cd treatment result in docetaxel exposure in the skin causing cutaneous anti-mitotic effects such as micronucleation, which could induce an inflammatory reaction leading to skin toxicity.

## 1. Introduction

Docetaxel is a potent anticancer drug [1] that is accompanied by serious side effects [2,3]. Docetaxel is indicated for the treatment of various stages of cancer in a variety of cancer types including breast cancer, non-small cell lung cancer, stomach cancer and prostate cancer. Because of its broad indications, it is one of the most prescribed chemotherapeutic drugs [4]. The antitumour activity of docetaxel is based on stabilisation of the microtubule dynamics, and thereby, disruption of the cell cycle leading to cell death [1]. Other healthy proliferating cells are unfortunately also affected by docetaxel resulting in side effects such as bone marrow suppression, hair loss and stomatitis [3].

Nanotechnology is an attractive approach to improve drug delivery and target selection [5]. Water-soluble nanoparticles loaded with chemotherapy are closed reservoirs of the native anticancer drugs, which increases the molecular weight and solubility whereas it simultaneously decreases the distribution volume of the native anticancer drugs. As a result of the leaky vasculature and poor lymphatic drainage of the tumour microenvironment, these loaded nanoparticles can accumulate in the tumour. Healthy tissues are (relatively) spared because of the normal endothelial fenestrations, which limit the tissue penetration of a nanoparticle. This phenomenon is called the “enhanced permeability and retention effect (EPR)” [5,6,7]. Nanoparticles can be designed to carry one or more agents, release drugs via environment specific triggers like pH or enzymatic catalysis, or can be loaded with a combination of diagnostic and therapeutic agents [8].

CPC634 is a novel nanomedicine consisting of docetaxel covalently entrapped in stabilised, 65 nm sized core-cross linked polymeric micelles (CCL-PMs). In preclinical studies, CPC634 demonstrated enhanced pharmacokinetics and an improved therapeutic index [9]. To investigate if CPC634 also has an improved pharmacokinetic profile compared to the native drug docetaxel in the clinical setting, a randomised cross-over study (the CriTax study) was conducted comparing the plasma and the intratumoural drug concentration of CPC634 head-to-head with conventional docetaxel (Cd). Patients received CPC634 during treatment cycle 1 and Cd during treatment cycle 2 or vice versa. This study demonstrated that CPC634 enhanced the intratumoural total docetaxel exposure [10]. Nevertheless, in a phase 1 trial with CPC634 (the Napoly study), cumulative skin toxicity was the most prominently observed dose-limiting toxicity [11]. To unravel the mechanisms behind the cutaneous toxicity, we amended the CriTax study to include skin biopsies for explorative pharmacokinetic (PK) and pharmacodynamic (PD) analyses. The first objective of this study was to investigate the pharmacokinetics of Cd and CPC634 in the skin and its relation to the plasma exposure and the intratumoural docetaxel concentrations of both compounds. The second objective was to explore the histopathological/pharmacodynamic effects of both Cd and CPC634 in the skin. The results of these analyses are presented in the current manuscript.

## 2. Materials and Methods

### 2.1. Study Design and Patients

Adult patients with solid tumours for whom no standard therapy existed were included in this randomised two-armed pharmacokinetic cross-over study. Patients were eligible for study participation if the following criteria were met during screening: Eastern Cooperative Oncology Group (ECOG) performance status of 0 or 1, no unresolved grade >2 toxicities related to previous received therapies, adequate bone marrow function, adequate liver function, and adequate renal function. Intake of herbal or medicinal products that strongly induce or inhibit CYP3A4 was prohibited. Patients were randomised to receive CPC634 during treatment cycle 1 and Cd during treatment cycle 2 (arm A), or vice versa (arm B). Both drugs were administered intravenously at a dose of 75 mg/m^2^. The duration of a treatment cycle was 4 weeks for CPC634 and 3 weeks for Cd. Corticosteroid premedication was given before Cd infusion (3 × 8 mg oral dexamethasone, 12, 3, and 1 h before infusion). No premedication was administered before infusion of CPC634. This study was conducted in accordance with International Conference on Harmonization Good Clinical Practice guidelines, all applicable regulations and guidelines governing clinical study conduct, and ethical principles that have their origin in the Declaration of Helsinki. The study protocol was reviewed and approved by the Ethics Committee of Erasmus MC, Rotterdam. All patients gave written informed consent. This trial was registered in the Netherlands Trial Register (https://www.trialregister.nl/trial/6299 (accessed on 23 July 2021)).

### 2.2. Skin Biopsies

Punched skin biopsies of 3 mm from the arm were obtained under local anaesthesia with lidocaine 2% at baseline (PD biopsy) and at day 8 (biopsies for PK and PD) of both treatment cycles. Similar sampling locations were selected for the two treatment cycles. For PK analysis, a plasma sample was also taken at day 8 of both cycles.

### 2.3. Pharmacokinetic Analysis

Skin biopsies used for PK analysis were directly stored at ≤−70 °C until further analysis. Detailed description of the time points for plasma and tumour sampling are described in the published manuscript of the CiTax study [10]. All the samples (skin, tumour and plasma) were analysed using a validated liquid chromatography-mass spectrometry method as previously described [12]. Before docetaxel measurements, skin biopsies were homogenised using a tissue-lyser (Qiagen, Hilden, Germany) and a stainless-steel bead for 90 s at 60 Hz in 400 µL of blank plasma. Pharmacokinetic analysis included measurement of released docetaxel and total docetaxel (released docetaxel plus a docetaxel reservoir covalently bound to the nanoparticles). Released docetaxel from CPC634 nanoparticles was determined in human plasma stabilised with 5 M ammonium acetate, pH 5.0. Total docetaxel was determined by incubation of human plasma with 0.5 M ammonium acetate buffer pH 7.4 for 3 days at 37 °C to ensure the release of all the entrapped docetaxel from the nanoparticle for quantification. The validated ranges were 0.250–100 ng/mL for released docetaxel, and 2000–100,000 ng/mL for the higher concentrations and 2–500 ng/mL for the lower concentrations of total docetaxel.

### 2.4. Histopathological Analysis

Skin biopsies taken for histopathological assessments were directly fixed in formalin. Formalin-fixed paraffin embedded (FFPE) tissue sections were stained with hematoxylin and eosin for light microscopic evaluation using standard procedures. The total number of apoptotic bodies and mitotic figures were counted per section. Keratinocyte micronucleation, keratinocyte nuclear atypia and dermal inflammation were scored on a semi-quantitative scale from 0–3 (0 absent; 1 mild; 2 moderate; 3 profound). The percentage of Ki-67 positive keratinocytes was estimated as a marker of proliferation. Immunohistochemistry for Ki-67 was performed with an automated, validated and accredited staining system (Ventana Benchmark ULTRA, Ventana Medical Systems, Tucsen, AZ, USA) using ultraview universal DAB detection Kit. In brief, following deparaffinisation and heat-induced antigen retrieval the tissue samples were incubated according to their optimised time with the antibody of interest (Table S1). Incubation was followed by hematoxylin II counter stain for 12 min and then a blue colouring reagent for 8 min according to the manufacturer’s instructions (Ventana). To evaluate the structure of the skin capillaries, CD31 and SMA staining was done by automated multiplex IF using the Ventana Benchmark Discovery (Ventana Medical Systems Inc. Oro Valley, Arizona, United States of America). In brief, following deparaffinisation and heat-induced antigen retrieval with CC1 (#950–500, Ventana) for 32 min the tissue samples were incubated firstly with CD31 for 32 min at 37 °C followed by detection with Red610 (#760–245, Ventana). The antibody denature step was performed using CC2 (#950–123, Ventana) for 20 min at 100 °C. Secondly, SMA was incubated for 32 min at 37 °C followed by detection with FAM (#760–243, Ventana). Slides were incubated with DAPI in PBS and covered with anti-fading medium (DAKO, S3023). Antibody information and clonality can be found in the Supplementary Materials, Table S1.

### 2.5. Statistics

Differences in docetaxel concentration in the skin after both treatment cycles were estimated by means of a linear mixed effect model with treatment, sequence, and period as fixed effects and subject within sequence as a random effect on log-transformed data. Restricted maximum likelihood (REML) methods were used for estimation of variance components, and the Kenward–Roger method was used for computing the denominator degrees of freedom [13]. Exponentiation of the results including the 95% confidence interval (CI) results in the geometric mean ratio and its 95%CI. Intratumoural and skin concentration of docetaxel were compared using a paired *t*-test. The relation between skin, tumour and plasma PK (peak plasma concentration (*C*_max_), area under the curve (AUC) were explored by Pearson’s correlations [10].

## 3. Results

Skin biopsies for PK analysis were taken from 27 patients of whom 18 patients underwent paired biopsies on day 8 and two patients on day 15 due to logistical reasons. Of these, 10 patients also underwent paired biopsies for PD assessment.

### 3.1. Pharmacokinetics

**Figure 1:**
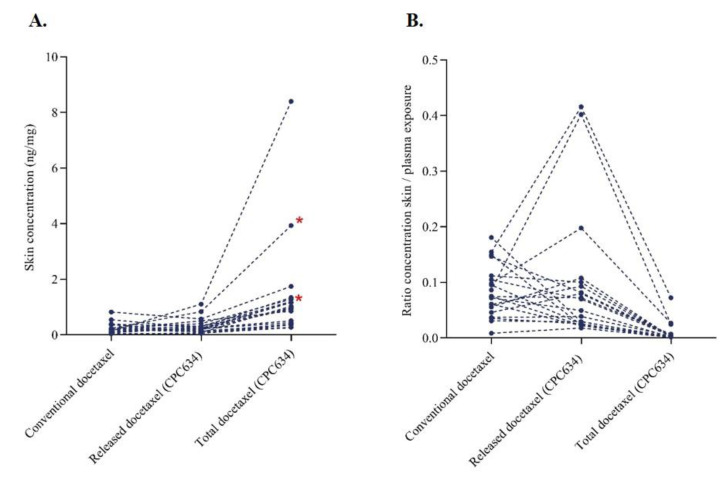
Docetaxel pharmacokinetics in the skin. (**A**) Docetaxel concentration in the skin after conventional docetaxel and CPC634 (released and total) administration (*n* = 20). (**B**) Docetaxel concentration ratio in the skin relative to plasma docetaxel concentration. * In these patients, skin biopsies were taken on day 15 of both cycles (*n* = 2).

The geometric mean (GEM) total docetaxel concentration in the skin of 0.89 ng/mg (95%CI: 0.59 ng/mg to 1.34 ng/mg) was 369% (95%CI: 229% to 569%, *p* < 0.001) higher after CPC634 administration compared to the GEM total docetaxel concentration in the skin of 0.19 ng/mg (95%CI: 0.13 ng/mg to 0.27 ng/mg) after Cd administration. In contrast, the released concentration in the skin after CPC634 (GEM: 0.23, 95%CI: 0.16 ng/mg to 0.34 ng/mg) was not statistically different (relative difference of 22%, 95%CI −9% to 63%, *p* = 0.169) compared to the observed docetaxel concentration (GEM: 0.19 ng/mg, 95%CI: 0.13 ng/mg to 0.27 ng/mg) after Cd infusion (Figure 1A). Docetaxel concentrations in the skin relative to plasma docetaxel concentration (Figure 1B) and intratumoural concentration (Table 1) were lower for both Cd and CPC634. A positive correlation (r = 0.477, *p* = 0.034) was found between released docetaxel plasma AUC_inf_ and released docetaxel in the skin (Table 2).

### 3.2. Histopathological Evaluation

**Figure 2:**
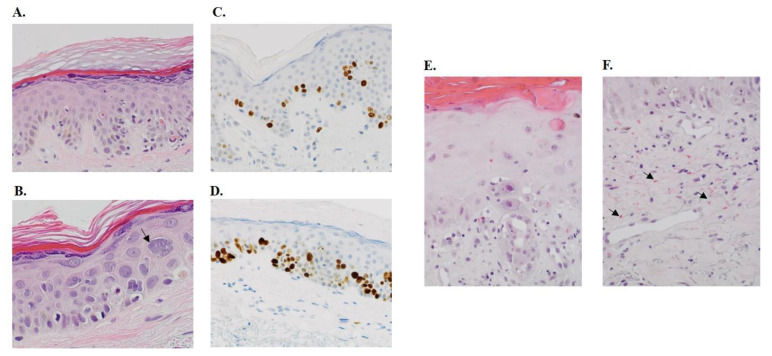
Histopathological changes in the skin. (**A**) This shows a skin biopsy of a patient after treatment with CPC634 with epidermis demonstrating hypergranulosis, influx of lymphocytes, basal vacuolisation of keratinocytes and scattered apoptotic cells along the dermo-epidermal junction (20× magnification). (**B**) This section is a 40× magnification of the same patient showing keratinocyte micronucleation (“grape cells”, arrow), also notice the accompanying apoptosis. Immunohistochemical stain for Ki67 at baseline (**C**) and after CPC634 treatment (**D**) (20× magnification). (**E**) This skin biopsy section of a patient in the Napoly study with skin toxicity shows an epidermis demonstrating vacuolar interface dermatitis with basal vacuolisation, influx of lymphocytes, scattered high apoptosis and hyper-and parakeratosis (20× magnification). Note the keratinocyte atypia with polymorphic nuclei and prominent nucleoli. (**F**) This section of the same patient shows ectatic capillaries and postcapillary venules of the superfical vascular plexus, a minor lymphohistiocytic perivascular infiltrate and extensive erytrocyte extravasation (arrow) (20× magnification).

**Figure 3:**
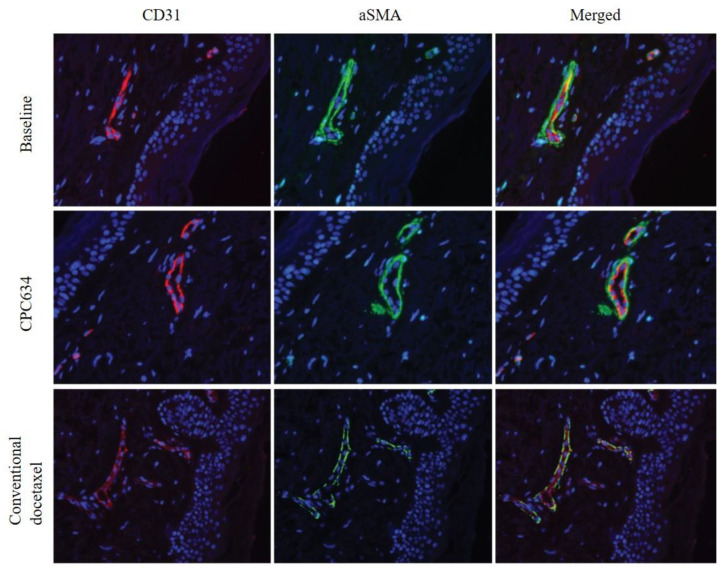
CD31 and aSMA staining of the skin biopsies at baseline, after administration of CPC634 and conventional docetaxel.

After both treatment cycles, skin biopsies revealed an increased number of apoptotic keratinocytes, a higher number of mitotic cells, keratinocyte atypia and keratinocyte micronucleation (Figure 2A–D, Table 3). The skin biopsy from a patient with severe skin toxicity in the Napoly trial who received 2 CPC634 cycles at 80 mg/m^2^ without premedication showed a profound epidermal hyperplasia, hypergranulosis, hyperkeratosis, nuclear hyperchromasia, high apoptotic cell and erythrocyte extravasation, indicating capillary damage (Figure 2E–D). CD31 and aSMA staining of the skin biopsies in the CriTax study demonstrated preserved skin capillaries during both treatments (Figure 3).

## 4. Discussion

CPC634 has demonstrated enhanced intratumoural total docetaxel exposure compared to Cd, resulting from the EPR effect and a lower incidence of neutropenia presumably related to lower plasma *C*_max_ of released docetaxel by CPC634. Cumulative skin toxicity was an unexpected dose-limiting toxicity that resembled Cd-induced skin toxicity in the phase 1 Napoly trial of CPC634 [11,14,15]. The underlying mechanism of docetaxel-related skin toxicity has never been related to the pharmacology of this drug in the skin before. This is the first clinical study to assess docetaxel exposure in the skin after Cd and CPC634 infusion. Cd-induced skin toxicity has been described to include maculopapular rash or eruptions, palmar-plantar erythrodysaesthesia, (recall) dermatitis, immediate hypersensitivity reaction, drug-induced lupus erythematosus, photosensitivity, pustular eruptions, pigmentary and scleroderma-like changes [16]. The incidence of Cd-induced skin toxicity varies widely across different publications, depending on the intensity of the administered docetaxel dose, the cumulative dose, frequency and premedication regimen [14]. The incidence of all grade Cd-related skin toxicity has been reported to be between 6% and 67% with docetaxel monotherapy preceded by corticosteroid premedication [14,17]. In early studies with Cd monotherapy without corticosteroid premedication, the incidence of grade ≥3 skin toxicity has been reported to be even as high as 70% [18]. In the Napoly trial, skin toxicity was the most prevalent grade ≥3 toxicity, which occurred in 24% of the patients.

Here, we showed that after CPC634 administration without corticosteroid premedication, the exposure to total docetaxel in the skin is almost four-fold higher compared to Cd. The conserved docetaxel in the nanoparticles is assumed to release over time ensuring a prolonged released docetaxel exposure in the tumour [9]. We hypothesise that CPC634-induced cumulative skin toxicity is related to a prolonged release exposure in the skin that is intensified after repeated administrations. This is supported by the fact that the released docetaxel plasma AUC for CPC634 is higher compared to that of Cd [10] and a positive correlation is observed between released docetaxel plasma AUC and released docetaxel concentration in the skin. Furthermore, the lower docetaxel concentrations in the skin compared to the plasma docetaxel concentrations and the intratumoural docetaxel concentrations demonstrate that the EPR effect is likely more profound in the tumour. Nevertheless, incomplete clearance of released docetaxel from the skin due to a short treatment interval and repeated high dose infusions of CPC634 could induce capillary damage, causing an advanced EPR effect in the skin as well as resulting in higher and longer docetaxel concentration and aggravating skin toxicity. Interestingly, in the CriTax study, no severe skin toxicity was observed. We propose that this results from the preserved walls of capillaries in the skin, which prevent further extravasation of the CPC634 nanoparticles in the skin after only one treatment cycle of CPC634. Analysis of skin biopsies from a patient with grade ≥3 skin toxicity in the phase 1 Napoly trial with CPC634 revealed extravasation of erythrocytes in the affected regions that indicate damage of the skin capillaries. Prolonging the infusion interval potentiates sufficient docetaxel clearance, thereby preserving skin capillaries [19]. Based on the higher and prolonged intratumoural docetaxel concentration of CPC634 compared to Cd [10], one could also propose to investigate whether lower dose administration of CPC634 would be as effective as the registered conventional dose while limiting the adverse events of CPC634.

Histopathological evaluation of the non-affected skin biopsies showed that both CPCP634 and Cd revealed increased anti-mitotic effects such as micronucleation. Micronucleation results from mitotic chromosome segregation errors, leading to partitioning of the genome into many small nuclei [20]. It is proposed that inflammatory signalling, caused by micronucleation, plays a vital role in the anti-tumour response of taxanes [20]. We hypothesise that this inflammatory reaction also occurs in the skin due to prolonged docetaxel exposure, resulting in skin toxicity. Dexamethasone premedication improves skin tolerability of CPC634 [11], most likely as a result of its anti-inflammatory effects [19]. This is presumably also the reason for the decreased incidence of severe skin toxicity after introduction of corticosteroid premedication with Cd administration in the Napoly trial.

The main limitation of this study was that patients received only one CPC634 and one Cd treatment cycle. Thus, the effect of repeated administration of CPC634 and Cd on the skin could not be assessed in this study. A randomised double blinded cross-over trial in which patients receive repeated treatment cycles is needed to confirm the current results and our proposed hypothesis regarding the role of prolonged exposure of released docetaxel in the skin and micronucleation to induce inflammation in the skin. This study demonstrates that insight into the pharmacokinetics of a drug in different types of tissue is indispensable to understand the pharmacodynamic effects related to adverse events. These results also highlight that it is important to demonstrate a pharmacokinetic-toxicity relation for anticancer drugs and to explore strategies to modify the pharmacokinetic profile of these drugs to improve their tolerability.

## 5. Conclusions

In conclusion, CPC634 and conventional docetaxel treatment both result in docetaxel exposure in the skin causing micronucleation that could induce an inflammatory reaction resulting in skin toxicity.

## Figures and Tables

**Table 1:** Intratumoural docetaxel difference (for both CPC634 and conventional docetaxel) relative to the skin.

	Geometric Mean Ratio	95% Confidence Interval	Significance (2-Tailed)
Tumour TDx vs. skin TDx	3.501	2.197–5.579	<0.001
Tumour RDx vs. skin RDx	2.918	1.960–4.345	<0.001
Tumour Cd vs. skin Cd	3.133	1.745–5.618	0.001

Abbreviations; total docetaxel (TDx), released docetaxel (RDx), conventional docetaxel (Cd).

**Table 2:** Pearson’s correlation for docetaxel concentration in the tumour versus the skin; plasma docetaxel pharmacokinetics versus skin docetaxel concentrations.

	Pearson’s Correlation	Significance (2-Tailed)
Tumour TDx vs. skin TDx	0.224	0.342
Tumour RDx vs. skin RDx	0.220	0.351
Tumour Cd vs. skin Cd	0.110	0.663
AUC_inf_ TDx vs. skin TDx	0.351	0.129
AUC_inf_ RDx vs. skin RDx	0.477	0.034
AUC_inf_ Cd vs. skin Cd	0.441	0.052
*C*_max_ TDx vs. skin TDx	0.322	0.166
*C*_max_ RDx vs. skin RDx	0.288	0.218
*C*_max_ Cd vs. skin Cd	0.478	0.033

Abbreviations; total docetaxel (TDx), released docetaxel (RDx), conventional docetaxel (Cd).

**Table 3:** Semi-quantitative histopathological analysis of the skin biopsies (0 absent; 1 mild, 2 moderate, 3 profound).

Treatment Arm	Apoptosis			Mitosis			Micronucleation			Atypia			Inflammation			Ki67		
	Baseline	CPC634	Cd	Baseline	CPC634	Cd	Baseline	CPC634	Cd	Baseline	CPC634	Cd	Baseline	CPC634	Cd	Baseline	CPC634	Cd
A	0	13	3	0	10	0	0	2	1	0	2	1	1	1	1	1%	50%	5%
A	0	11	3	1	4	4	0	1	1	0	1	1	1	1	1	5%	5%	5%
A	5	97	8	1	31	6	0	3	2	0	1	1	1	1	1	5%	50%	25%
A	1	9	4	0	7	1	0	1	1	0	1	0	1	1	1	5%	5%	5%
A	0	21	6	0	5	9	0	1	3	0	0	2	1	1	1	5%	5%	5%
A	0	9	26	0	3	8	0	1	2	0	1	1	1	1	1	5%	5%	50%
A	0	7	NA	0	2	NA	0	1	NA	0	1	NA	1	1	NA	5%	5%	NA
B	0	0	8	0	4	0	0	2	1	0	1	1	1	1	1	5%	5%	25%
B	0	1	0	1	0	1	0	0	0	0	0	0	1	1	1	5%	5%	5%
B	0	12	8	3	3	0	0	2	1	0	1	1	1	1	1	5%	50%	5%

Abbreviations; conventional docetaxel (Cd); not applicable (NA).

## Data Availability

The datasets used during the current study are available from the corresponding author on reasonable request.

## Supplementary Materials

The following are available online at https://www.mdpi.com/article/10.3390/cancers13153741/s1, Table S1: Antibody information for Ki-67, CD31 and SMA staining.
