# Supplementary material for: Docetaxel Skin Exposure and Micronucleation Contributes to Skin Toxicity Caused by CPC634
Source: Cancers (Basel). 2021 Jul 26;13(15):3741. doi: 10.3390/cancers13153741 (PMC8345028; doi:10.3390/cancers13153741)
Supplement: Supplementary file 1 [file cancers-13-03741-s001.zip › cancers-1264735-supplementary.pdf]

Atrafi et al: Docetaxel-induced skin toxicity is caused by micronucleation

Supplementary file

Table S1. Antibody information for Ki-67, CD31 and SMA staining

Antibody	Type	Concentration	Company	Clone
Ki-67	Anti-Rabbit	2.0 µg/mL	Ventana	30-9
CD31	Anti-Mouse	0.81 µg/mL	Ventana	JC-70
SMA	Anti-Mouse	0.02 µg/mL	Ventana	1A4
